# Brain gain—Is the cognitive performance of domestic hens affected by a functional polymorphism in the serotonin transporter gene?

**DOI:** 10.3389/fpsyg.2022.901022

**Published:** 2022-09-15

**Authors:** Anissa Dudde, Loc Phi Van, Lars Schrader, Arnd J. Obert, E. Tobias Krause

**Affiliations:** ^1^Institute of Animal Welfare and Animal Husbandry, Friedrich-Loeffler-Institut, Celle, Germany; ^2^Hannover Medical School, Institute for Diagnostic and Interventional Radiology, Hannover, Germany

**Keywords:** chicken cognition, learning, serotonin transporter, domestic chicken, reversal learning

## Abstract

The serotonin transporter (5-HTT) plays an important role in regulating serotonergic transmission *via* removal of serotonin (5-HT) from synaptic clefts. Alterations in 5-HTT expression and subsequent 5-HT transmission have been found to be associated with changes in behaviour, such as fearfulness or activity, in humans and other vertebrates. In humans, alterations in 5-HTT expression have been suggested to be able to lead to better learning performance, with more fearful persons being better at learning. Similar effects of the variation in the 5-HTT on fearfulness have been found in chickens, and in this study, we investigated effects on learning. Therefore, we tested 52 adult laying hens, differing in their functional 5-HTT genotype (W/W, W/D and D/D) in an operant learning paradigm in three different phases (initial learning, reversal learning and extinction) and in a tonic immobility test for fearfulness. We found that the 5-HTT polymorphism affects the initial learning performance of laying hens, with homogeneous wild-type (W/W) hens being the slowest learners, and the most fearful birds. W/W hens, showed significantly more choices to solve the initial learning task (LME, *p* = 0.031) and had the highest latencies in a tonic immobility test (*p* = 0.039), indicating the highest fearfulness. Our results provide interesting first insights into the role of 5-HTT in chickens and its sensitive interaction with the environment. We further suggest that the 5-HTT gene can be an interesting target gene for future breeding strategies as well as for further experimental studies.

## Introduction

Serotonin (5-hydroxytryptamine, 5-HT) is an important hormone in the body as well as a key regulatory neurotransmitter in the brains of vertebrates (Lesch et al., 1996). The transmission of neural serotonin from the pre-to the postsynapse is controlled through a reuptake of 5-HT from the synaptic clefts by the serotonin transporter (5-HTT, abbreviation for the 5-HT transporter). Thus, serotonin transporters are crucially involved in the neural serotonin regulatory system. In humans, a polymorphism of the 5-HTT gene is associated with functional consequences in the emotional system (Lesch et al., 1996). Humans with an S-allele, i.e. a short allele, in comparison to the individuals with an L-allele, i.e. long allele, have a lower 5-HTT expression as a result of decreased 5-HTT gene promoter efficiency. This change contributes to behavioural modifications, such as increased anxiousness-like traits or the prevalence of the occurrence of depression (Lesch et al., 1996; Canli and Lesch, 2007). Similar patterns are also found in other mammalian species, such as monkeys (Lesch et al., 1997) and rodents (Brigman et al., 2010).

These effects of polymorphisms in the 5-HTT gene on behaviour have led the focus to similar effects in other mammals. The domestic chicken possesses a functional polymorphism in the 5-HTT gene (Phi-van et al., 2014), with a wild-type allele (W) and a deletion allele (D). In the deletion allele, four bases are deleted (5′-AATT-3′), and a single base (A → T) is exchanged (Phi-van et al., 2014) in comparison to the wild-type allele. Interestingly, the W allele of domestic chickens functionally resembles the human S allele in terms of lowered 5-HTT expression and increased fear-like behaviours (Phi-van et al., 2014; Krause et al., 2017; Phi Van et al., 2018). In line with this behaviour, the D-allele of domestic chickens shows 5-HTT and behavioural pattern expression comparable to the human L-allele. Domestic chickens with D-alleles, in comparison to homozygous W-allele chickens, show increased body mass and abdominal fat deposition (Phi-van et al., 2014; Kjaer and Phi van, 2016; Krause et al., 2017), increased feed uptake during ontogeny (Kjaer and Phi van, 2016), increased locomotor activity (Phi-van et al., 2014; Krause et al., 2019), and a lower level of fearfulness (Krause et al., 2017, 2019) but no differences in social-related behaviours (Krause et al., 2019).

One aspect, linked to the polymorphism at the 5-HTT gene in humans, has not yet been studied in domestic chickens. In humans, 5-HTT polymorphisms are thought to be linked to cognitive performance, such as learning (Lesch et al., 1997). The S-allele human carriers tend to show increased attention towards biological conditioned stimuli and hence might perform better in learning (Homberg and Lesch, 2011). This assumption is supported by other studies showing that humans carrying S-alleles perform better in decision-making and learning than homozygote L-allele carriers (Roiser et al., 2007; Strobel et al., 2007; Madsen et al., 2011; Karabeg et al., 2013). In line with these findings, mice and monkeys demonstrated a higher flexibility in learning when carrying shorter alleles in various reversal learning tasks using visual or auditorial cues (Brigman et al., 2010; Jedema et al., 2010; Harris et al., 2012). However, in rats, such differences were not revealed (Karabeg et al., 2013). Based on the strong indications of a role of 5-HTT polymorphisms in cognitive performance in mammalian species, we aimed to address the question of whether the domestic chicken polymorphism in the 5-HTT gene also has a functional impact on cognitive performance.

Complex forms of learning are in general quite well documented in domestic chickens (Krause et al., 2006; Marino, 2017; Dudde et al., 2018; Garnham and Løvlie, 2018). Thus, we aimed to characterise the cognitive abilities of domestic chickens with three different 5-HTT genotypes, homozygous wild-type W/W, homozygous deletion D/D and heterozygous genotype W/D, and to validate their differences in fearfulness using a tonic immobility test (Krause et al., 2019). To study their cognitive performance, we used an established automated operant learning paradigm for domestic chickens (Dudde et al., 2018), which included initial associative learning, followed by reversal learning and finally an extinction phase. In accordance with mammalian studies and the convergence of the human S-allele with the domestic chicken W-allele, we assume that domestic chickens with a homozygous wild-type allele W/W would perform better in the cognitive task and be more fearful compared to the homozygous deletion D/D birds, while heterozygous chickens should perform in the intermediate range.

## Materials and methods

### Animals and housing

Adult domestic hens (*Gallus gallus* forma domestica) with polymorphisms in the flanking region of the 5-HTT gene were used (Phi-van et al., 2014). The D-allele is characterised by the deletion of four nucleotides (5′-AATT-3′) and a nearby single nucleotide substitution (A → T) compared to the wild-type allele W (Phi-van et al., 2014; Phi Van et al., 2018). From these two alleles, three 5-HTT genotypes appeared: homozygous wild-type W/W, homozygous deletion-type D/D and heterozygous W/D. The hens with the three 5-HTT genotypes were obtained through a controlled breeding regime using W/W and D/D parents from the laboratory stock, returning to the genetic Lohmann Brown (Phi-van et al., 2014; Kjaer and Phivan, 2016; Krause et al., 2017; Phi Van et al., 2018). Briefly, 20 cockerels of each genotype were randomly intercrossed with 20 hens of the same genotype and with 10 hens of the other genotype. The hens of that breeding were marked with numbered wing tags and raised in identical littered pens until the experiments. For the experiment, we used 52 hens, 15 hens of the W/W, 19 hens of the W/D and 18 hens of the D/D alleles that had an age of 1.5 years at the start of the tests. The three genotypes of the hens are not linked to their phenotypic appearance, which enabled us to conduct all data collection blind with respect to the genotype of the animal. The genotypes were revealed after completion of data collection for the analysis.

Approximately 12 weeks before the behavioural tests started, hens were randomly allocated to two identical compartments that were next to the room used for the behaviour test, all in the same stable. These litter floor compartments (each a size of 11 m^2^) were equipped with perches, a box filled with sand for dustbathing, pick blocks and a group nest. The birds had *ad libitum* access to standard commercial food (15.5% crude protein, 5.2% crude fat, 3.4% crude fibre, and 12.8% crude ash; the three main ingredients were 35.7% wheat, 18.4% maize, and 17.3% soy) and to water. Light was provided for 14 h per day. To habituate the hens to the experimenters and the food reward of the test, the chickens were additionally fed wheat grains once a day by the experimenters.

### Learning experiment setup

The experimental setup and the procedures of the learning experiment were similar to the experimental setup and the procedures described in Dudde et al. (2018). The procedures are briefly described in the following. Hens were trained and tested in different phases: (a) habituation, (b) screen training that consisted of three stages, and (c) the cognitive test phase that consisted of three learning stages: (i) initial associative learning, (ii) reversal learning, and (iii) extinction (see Table 1). The hens were tested during these phases in one out of four identical custom-built test boxes, which were located in a room adjacent to the home compartments of the hens. The test boxes (width, depth, height: 55 cm × 46.5 cm × 66 cm) with a touchscreen (height × wide: 19 cm × 25 cm), a speaker and a food reward-delivery system (foldable food trough (height × wide × depth: 1.5 cm × 4 cm × 8 cm)) are described in detail in Dudde et al. (2018). An in-house developed C++ software (Microsoft Visual Studio, 2010, Microsoft, Redmond, WA, United States; code can be provided upon request) controlled the complete electronic setup of the test box, such as light, sound, reward delivery, touchscreen and monitor. For additional observation of the hens, a video camera was installed at one side of each test box. During the experiments, the hens were not able to see each other from inside the boxes. For habituation, training and learning phases, hens were individually taken from their home compartments and gently placed into the test boxes. They were rewarded with wheat grains to which they had been familiarised in advance. At no time in the experiment were hens’ food restricted prior to testing. The experience of success with only positive rewards was established in a previous study (Dudde et al., 2018). The time in the test box was increased during the habituation stage (see Table 1). The hens remained in the test box for a session that lasted up to a maximum of 20 min. However, if a hen made quicker decisions, she could decrease the time in the test box in that respective session, as each hen had to make 20 decisions per session (for details see Dudde et al. (2018)) or alternatively, the time in the test box ended after 20 min. If a hen did not finish one of the training phases (see details below) or one of the three learning phases within 20 daily sessions, the testing ended, and she was thus excluded from the further experiments (Dudde et al., 2018). To successfully solve each training phase, initial learning and reversal learning, hens needed 80% correct decisions out of at least ten decisions (Dudde et al., 2018). This learning criterion differs from the 50% chance level and is in accordance with other learning studies (Garner et al., 2006; Brust et al., 2014; Dudde et al., 2018). To successfully finish the extinction, hens needed to demonstrate no responses in 70% of at least ten trials (Dudde et al., 2018).

**Table 1:** Experimental phases and their characteristics.

	Level	Time	Stimulus	Task/reward for	Learning criteria
Habituation	0	One session	None	Stay 5 min in the box and eat wheat grain *ad libitum*	None
		One session	None	Stay 10 min in the box and eat wheat grain *ad libitum*	None
		One session	None	Stay 10 min in the box and eat wheat grain *ad libitum*, turning on and off of reward delivery system	None
		One session	None	Stay 15 min in the box and eat wheat grain only when reward system turns on, time to eat 20 s	None
		One session	None	Stay 15 min in the box and eat wheat grain only when reward system turns on, time to eat 5 s	None
Screen training	1	Individual	Circle	Peck on circle or no peck on circle within 30 s—rewarded	80% correct
	2	Individual	Circle	Peck three times on circle—rewarded	80% correct
	3	Individual	Circle	Peck five times on circle—rewarded	80% correct
Discrimination	4	Individual	Bars	Peck five times on correct symbol—rewarded	80% correct
Reversal	5	Individual	Bars	Peck five times on correct symbol—rewarded	80% correct
Extinction	6	Individual	Bars	No response, not rewarded	70% correct

### Habituation and training phases

During the habituation phase, hens were individually habituated in five consecutive sessions, with increasing time in the box from 5 to 20 min (Table 1). Thereafter, the training phase started, and hens were familiarised with pecks on a dot on the touch screen to receive a food reward (Table 1). During training, hens were asked to go through three training phases as described in detail in Dudde et al. (2018). Once a hen had successfully finished the training, she was tested in the initial associative learning phase.

### Testing in three learning phases

The cognitive testing consisted of three phases: (i) initial associative learning, (ii) reversal learning, and (iii) extinction phase.

- Initial associative learning phase

For the initial associative learning, the hens needed to learn to differentiate between two simultaneously shown coloured bars, red and green, independent of the orientation of the bars (for details, see Dudde et al., 2018). Which colour was rewarded was alternatingly changed between subjects. To avoid side preferences, the presentation side of the rewarded bar was randomised on both sides within sessions (de Haas et al., 2017a,b). Pecking one of the bars was recorded as an active decision and as correct when the bar pecked was the rewarded bar and as incorrect when the bar packed was the unrewarded bar. Pecking the rest of the screen outside the bars, i.e. the black screen, was not recorded as a decision. If a hen made a correct decision, she received wheat grains for 5 s before the next trial appeared. Thereafter, a black screen was shown for 20 s (intercomponent time) before the two coloured bars reappeared with a randomised position and orientation. If a hen made a wrong decision, no reward was provided, and a black screen appeared for 5 s, followed again by 20 s of intercomponent time. Then, the previously shown bars appeared at the same position again. Hens solved the initial learning phase when they made 80% correct decisions of at least ten decisions within a session.

- Reversal learning phase

During reversal learning, the initially unrewarded colour was rewarded, and the initially rewarded colour was unrewarded. Everything else remained identical to the process described in the initial learning phase (Dudde et al., 2018). The reversal learning was successfully finished after 80% correct decisions of at least ten decisions within a session.

- Extinction phase

In the extinction phase, none of the two bars were rewarded, and the extinction learning criterion was reached when a hen did not respond to any of the two symbols on the screen in 70% of a least ten trials within a session. If a hen did not peck, the symbols vanished after 20 s, followed by an intercomponent time of 20 s. If a hen pecked on one of the symbols on the touchscreen, the black screen appeared for an intercomponent time of 20 s (see as well Dudde et al., 2018, for details).

### Stimuli used in the training and cognitive phases

During the training phase, we used as stimulus a grey circle (diameter: 2 cm, colour in red-green-blue (RGB) values: R = 224, G = 224, B = 224) that was shown on the touch screen. During the cognitive testing, we used a green bar (high × length: 10 mm × 40 mm, colour in RGB values: R = 20, G = 184, B = 29) and a red bar (high × length: 10 mm × 40 mm, colour in RGB values: R = 237, G = 28, B = 36) as stimuli, which were presented on a black screen and were all suited for the visual physiology of the hens (Osorio et al., 1999). These stimuli had already been used successfully in former experiments (Dudde et al., 2018). The screens were thin-film transistor (TFT) screens 12.1″ with super video graphics array (SVGA) 600 * 800 with an infrared (IR) frame for touch detection (IR Touch-kit 121.-A301, Citron GmbH, Augsburg, Germany).

### Fear-related measures estimated by the tonic immobility test

We further measured a fear-related trait in advance using the tonic immobility paradigm to understand potential genotype-related links between fear-related behaviours (Krause et al., 2017, 2019) and cognitive performance. Therefore, all individuals were individually tested in a tonic immobility (TI) test approximately 8 weeks prior to the learning test. The TI is a robust measure for fearfulness in chickens (Gallup et al., 1971; Jones, 1986). A longer latency to rise reflects a higher level of fear (Gallup et al., 1971; Jones, 1986). The test was conducted as described earlier (Krause et al., 2019): briefly, hens were individually tested in an adjacent room using a V-shaped cradle. Birds were put on their backs into the cradle, and once they remained immobile for 10 s, the latency to rise was measured (maximal 600 s). Birds that did not remain immobile within three attempts were recorded with 0 s, which was only the case for a single bird (from the D/D genotype). Immediately after testing, birds were released into their respective home compartments.

### Statistical analysis

To analyse the participation success of the hens from the different genotypes, we first compared the proportion of hens that met the criterion for each phase of the screen training (three phases) and cognitive tasks (three phases). Thus, hens solving all tasks successfully achieved 6 phases in total. These numbers were tested for survival curve differences using the Gehan-Wilcox test.

Thereafter, we compared the learning performance of the hens with respect to their 5-HTT genotype. We analysed the sum of active decisions they needed to fulfil the learning criteria of each level. Active decisions were counted as the number of correct and wrong decisions, whereby inactive trials with no decisions were not counted, in line with Dudde et al. (2018). The respective data processing was performed using a custom-written MATLAB script (Matlab and Statistics Toolbox Release, 2017). The residuals of the average number of active decisions per learning phase were tested for normal distribution with Shapiro–Wilk tests and homogeneity of variances with Levene’s test. We analysed the respective data for each learning level with a linear mixed effects (LME) model. The active decisions at the respective level were analysed in linear mixed effects models with the explanatory factor 5-HTT genotype (3-level factor: W/W, W/D, D/D) and housing compartment as a random factor. For significant linear models, we calculated *post-hoc* pairwise *t*-test comparisons for genotypes. Body weight was analysed using an LME model as described above. In addition, body weights were correlated to the active choice from the three different learning levels to examine whether there was a linkage using Pearson correlations. Latencies from the tonic immobility test were not normally distributed even after transformation and thus analysed with the nonparametric Kruskal–Wallis test with regard to the 5-HTT genotype, as a pairwise *post-hoc* unpaired Wilcoxon test was used. Furthermore, tonic immobility test latencies were correlated with the active choice from the three different learning levels to examine whether there was a linkage using Spearman correlations.

All analyses were calculated with R 4.0.3 (R Core Team, 2019), the package nlme (Pinheiro et al., 2007) car (Fox, 2019) and survival (Therneau, 2020).

The raw data of the study are available from Dudde et al. (2022).

### Ethical note

Animals were visually controlled daily for health status. The study was in accordance with the German Laws and has been approved by the respective regional authority, the Lower Saxony State Office for Consumer Protection and Food Safety (LaVes) (# 33.19–42,502–04-18/2993). The hens were housed as laying hens after the tests had ended, and the eggs were marketed.

## Results

### Success in participating throughout the experiment

**Figure 1:**
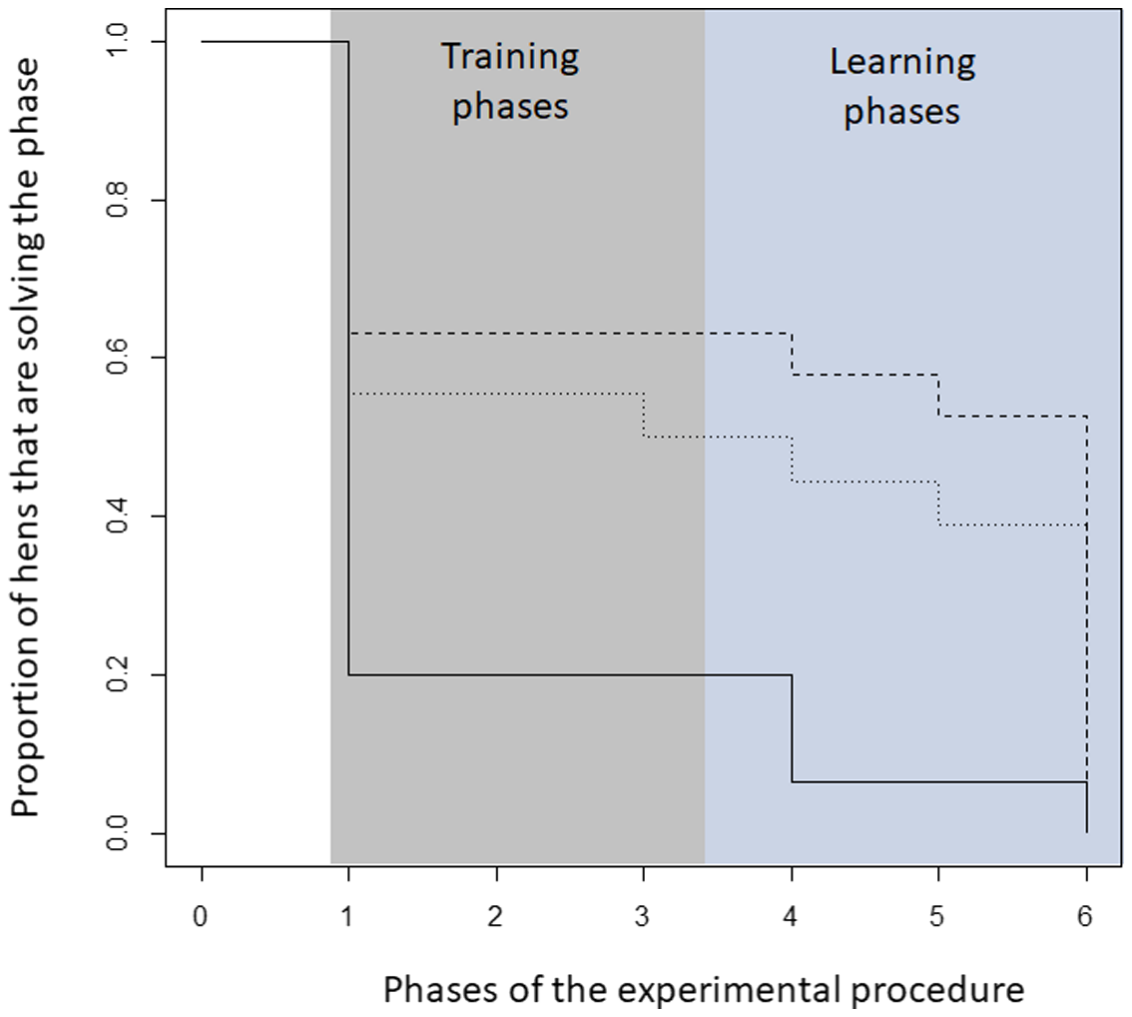
Proportion of hens that successfully passed the learning tasks of each level according to their genetic origin. In particular, hens from the W/W genotype failed to achieve the learning criteria of the different learning levels compared to hens from the D/D and W/D genotypes (Gehan-Wilcox test, Chi2 = 7.5, df = 2 *p* = 0.02). In the phases, the following number of hens took part in the respective phase: W/W (phase 1–6: 15,3,3,3,1,1); W/D (19,13,12,12,11,10); D/D (18,10,10,9,8,7).

The proportion of hens successfully participating in the experiment differed between the three 5-HTT genotypes. In particular, hens from the W/W genotype failed to achieve the learning criteria of the different learning levels throughout the experiment compared to hens from the D/D and W/D genotypes (Gehan-Wilcox test, Chi^2^ = 8.6, df = 2, *p* = 0.01, see Figure 1).

### Cognitive performance

- Initial learning

**Figure 2:**
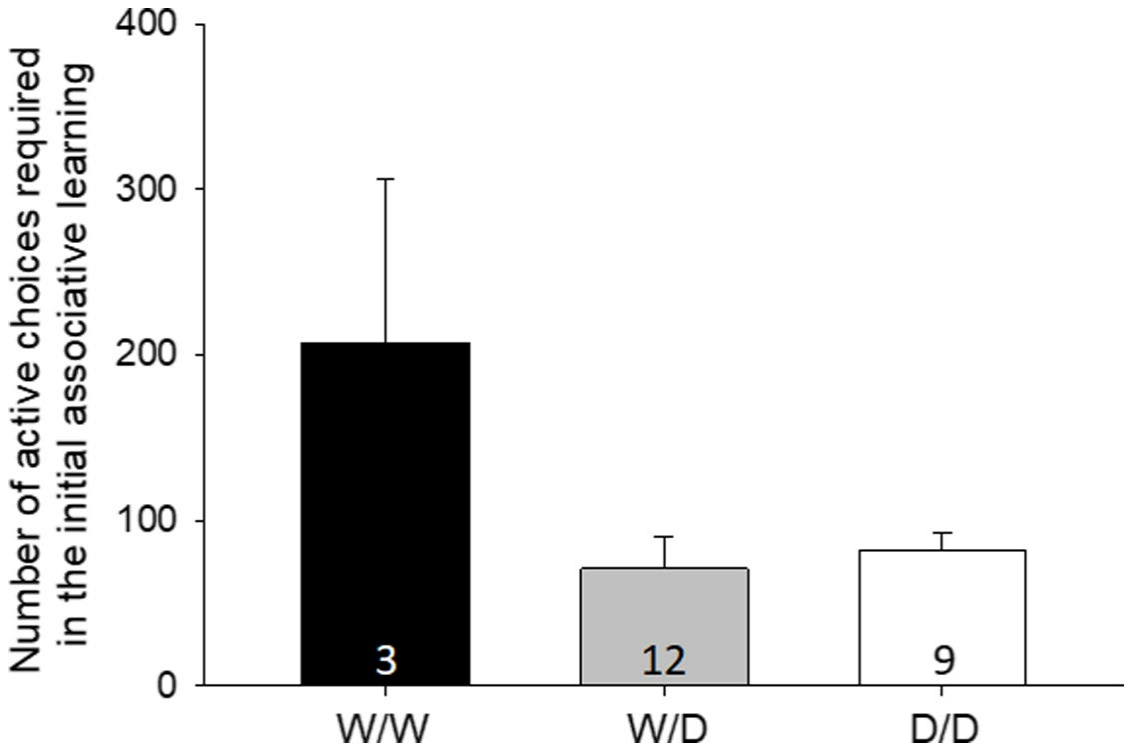
Mean number of active choices required in the initial learning [± standard error (SE)]. W/W hens needed more active decisions to reach the learning criteria compared to the other genotypes. The number in the bars indicates the number of hens that participated in the initial learning.

The initial learning performance was significantly affected by the 5-HTT genotype of the hens (LME: factor genotype: F_2,20_ = 4.14, *p* = 0.031, Figure 2). The *post-hoc* pairwise comparison revealed that W/W differed significantly from W/D and D/D (both *p* < 0.02), whereas D/D and W/D did not differ from each other (*p* = 0.73). W/W hens needed more active decisions to reach the learning criteria compared to the other genotypes. Three WW hens, 12 W/D hens and 9 D/D hens were tested in initial learning.

- Reversal learning and extinction

**Figure 3:**
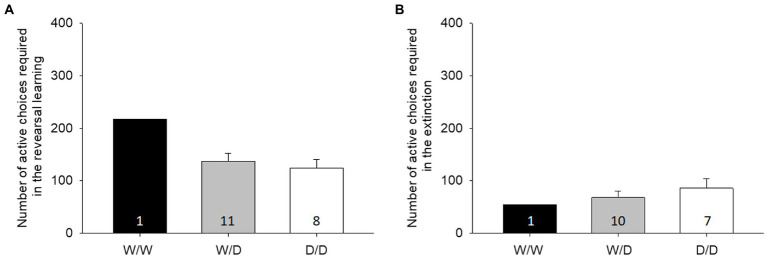
Mean number (± SE) of active choices required: **(A)** reversal learning, as well as **(B)** extinction learning, with no significant differences found between the three genotypes. The number in the bars indicates the number of hens that participated in the initial learning.

In reversal learning, hens from all three 5-HTT genotypes did not differ in the number of active decisions needed to reach the respective learning criteria (LME: factor genotype, F_2,16_ = 1.46, *p* = 0.26, Figure 3A). Additionally, in the extinction, 5-HTT genotypes did not show differences in the number of active choices (LME: factor genotype, F_2,14_ = 0.48, *p* = 0.63, Figure 3B). There were 1 W/W, 11 D/W and 8 D/D hens tested in the reversal tests and one less of each W/D and D/D in the extinction, according to the learning criteria.

### Body weight

**Figure 4:**
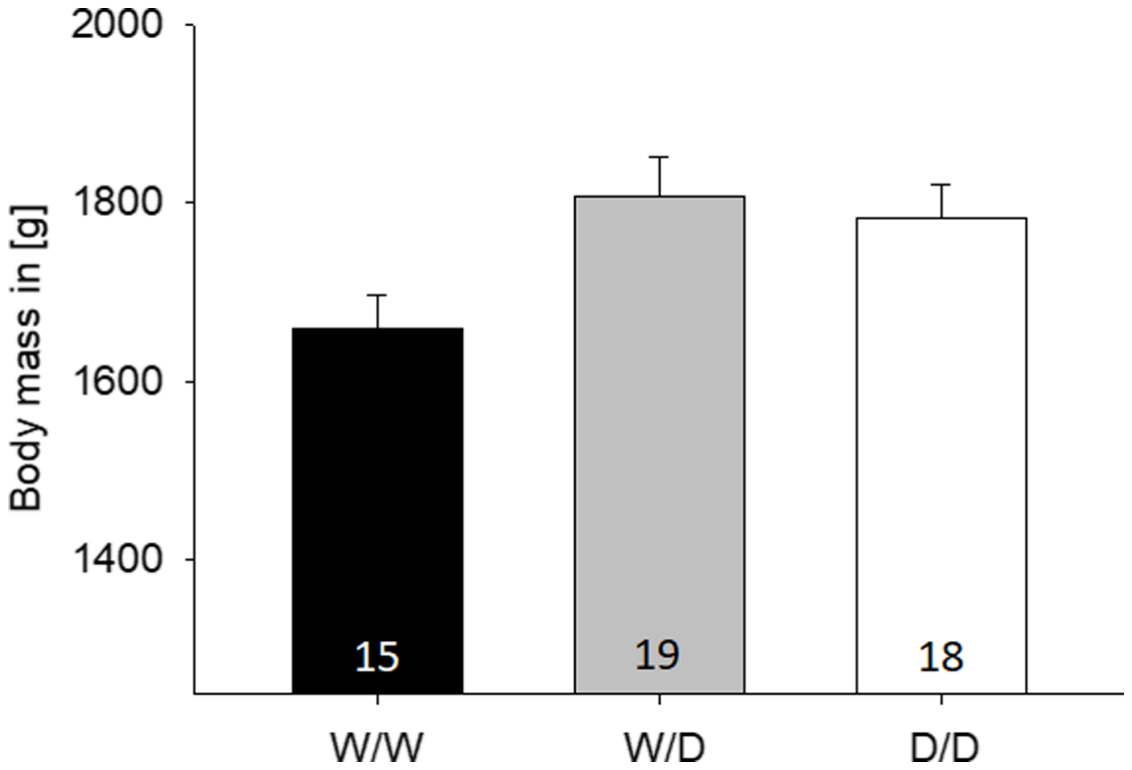
Mean body (±SE) mass in grams from the hens of the three 5-HTT genotypes, with the W/W being significantly lighter than the other two. The number in the bars indicates the number of hens that were measured.

The body weight of the hens from the three 5-HTT genotypes differed significantly from each other (LME: factor genotype, F_2,48_ = 4.60, *p* = 0.015; Figure 4). Pairwise *post-hoc* tests revealed that W/W differed from W/D and D/D (both *p* < 0.038), but D/D and W/D did not differ from each other (*p* = 0.65). On an individual level, body weight was not correlated with any of the three numbers of active choices from the cognitive performance (Pearson correlations, all three r < −0.16, all three *p* > 0.26).

### Fear-related measures estimated by the tonic immobility test

**Figure 5:**
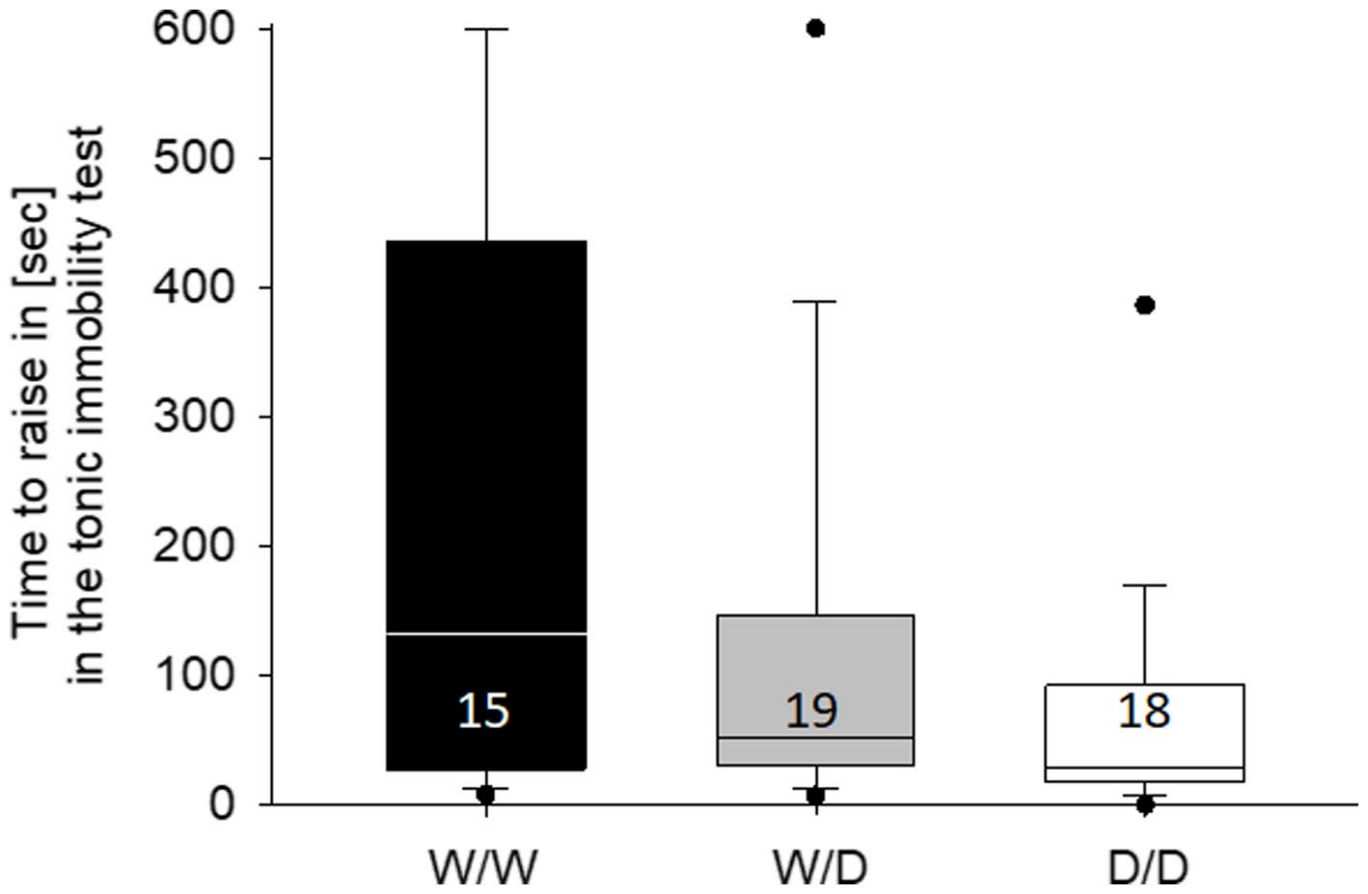
Median time in the tonic immobility test required by the hens of the three genotypes to rise up. W/W hens needed longer than the hens from the other genotypes. The number in the boxes indicates the number of hens that were measured.

The latency in the tonic immobility test differed between the hens of the three 5-HTT genotypes (Kruskal-Wallis test, X^2^ = 6.48, df = 2, *p* = 0.039; Figure 5). *Post-hoc* comparisons between the three 5-HTT genotypes showed that WW differed from DD (*p* = 0.018), while all other comparisons were not different (both *p* > 0.12). On an individual level, tonic immobility latency was not correlated with any of the three numbers of active choices from the cognitive performance (Spearman correlations, all three r < 0.25, all three *p* > 0.24).

## Discussion

Hens with the 5-HTT genotypes W/D and D/D performed significantly better in the initial learning phase than hens with the W/W genotype. The D/D and W/D hens not only performed better in the learning phase but also participated in all three cognitive phases at a higher rate than the W/W hens and were also less fearful in a tonic immobility test than the W/W hens.

This result on cognitive performance is in contrast to our initial assumption that was derived from humans and other mammalian studies (Homberg and Lesch, 2011); however, the result is in line with our expectation with regard to fearfulness (Krause et al., 2019). W/W hens were the most fearful, participated less in the learning task and had worse results in this task. This functional effect of the 5-HTT genotype is interesting. The effect may have been caused by several factors, which we discuss in the following.

One of the main factors affecting learning performance in this experiment can be the diverging fearfulness of the hens. The hens with the W/W genotype are more fearful than the other types (Krause et al., 2017, 2019), as shown in the tonic immobility test, where longer latencies to rise indicate a higher level of fearfulness (Gallup et al., 1971; Jones, 1986). This is an interesting difference from humans, where the genotype resembling the chicken W allele is the S allele, which is also linked to more fearful responses but is assumed to show more thoughtfulness towards learning tasks (Homberg and Lesch, 2011). In our experiment, an assumption for worse learning of D/D hens could be associated with the potentially stressful learning situation in the Skinner box, separated from the conspecifics, hence paying more attention to the environment but not to the actual learning task. Future studies should therefore also address the question of whether D/D and W/W hens differ in their coping style, e.g., reactive or proactive (Koolhaas et al., 1999, 2010), as such is also known to affect learning speed (Guillette et al., 2015). However, in contrast to humans, learning performance is not positively linked to fearfulness, but it might be that fear hinders chickens from training and learning. This idea also supports the finding that the participation success in training and testing over all experimental phases was significantly different between the genotypes and lowest in W/W hens, either reflecting their fearfulness or alternatively cognitive limitations.

Whether hens with the W/W genotype might be better at learning in a fear-free context might be considered in future studies. However, intrinsic fear cannot be compensated for by a fear-reducing environmental situation.

A further potential factor, which might theoretically have affected the learning performance of the hens in our study, is the different motivations for the food reward used in our test (Dudde et al., 2018). Previous studies, as well as this study, have shown that hens possessing the D/D genotype are heavier and feed more during certain juvenile stages in comparison to W/W hens (Kjaer and Phi van, 2016). Therefore, even adult D/D hens might have a higher food motivation in certain contexts and hence have a higher motivation to participate in the learning task. We cannot fully exclude effects caused by food motivation in this study design, although all hens were not food deprived prior to the study. Nevertheless, it could be interesting to design future experiments that use other than food rewards in cognitive tasks, e.g. social rewards, to avoid potential bias of food motivation.

Significant differences in cognitive performance were only detected in the initial learning, while during reversal, learning was solved by hens from all three 5-HTT genotypes similarly well. This finding has to be taken with a certain caution, as in the reversal and extinction phase, only a single hen from the W/W genotype participated in testing. Thus, whether the performance of the individuals from the three genotypes differs in these two phases cannot be robustly evaluated and may be further examined in future studies. Nevertheless, it is of great general interest that, although in the opposite direction as expected from mammalian studies, polymorphisms in the 5-HTT gene affect the initial learning of domestic chickens.

The results of the tonic immobility test show that W/W hens had the longest latencies to rise and thus the highest levels of fearfulness (Gallup et al., 1971; Jones, 1986). In addition to their help to potentially understand why W/W hens participated less and poorly in the learning test, the result itself is a nice replication of the findings of earlier studies (Krause et al., 2019). On the genotype group level, tonic immobility latencies were high in W/W hens as the number of active choices needed in the learning phases; however, no significant correlation between both parameters was found. Thus, there does not seem to be a strong association between the level of fearfulness and cognitive performance in individuals. No such correlation was found for body masses and cognitive performance; however, genotype-level differences in body masses replicate earlier studies (e.g. Phi-van et al., 2014; Krause et al., 2017, 2019).

From an applied animal science perspective, the polymorphism at the serotonin transporter gene is an interesting candidate for future commercial breeding strategies. Selecting for the deletion D-allele may lead not only to heavier hens but also to more robust and thus less fearful hens, which might be important for the mental welfare of the hens. However, thus far, the abundance of the D allele in commercial breeds investigated is quite low, e.g., approximately 7.5% in a brown layer strain (Krause et al., 2019).

Taken together, we found that polymorphisms in the serotonin transporter gene 5-HTT significantly affected the training and initial learning performance of laying hens. Genotypes related to less fearfulness perform better in the initial associative learning task, showing the impact of the 5-HTT polymorphism on cognitive performance in domestic chickens.

## Data availability statement

The original contributions presented in the study are publicly available. This data can be found here: https://doi.org/10.6084/m9.figshare.20237754.v1.

## Ethics statement

The study was carried out in accordance to the German Laws and has been approved by the respective regional authority, the Lower Saxony State Office for Consumer Protection and Food Safety (LaVes;# 33.19–42502–04-18/2993).

## Author contributions

AD, EK, LS, and LP contributed to conception and design of the study. AD and EK organised the experiments. EK and AO performed the statistical analysis. AD wrote the first draft of the manuscript. All authors contributed to the article and approved the submitted version.

## Conflict of interest

The authors declare that the research was conducted in the absence of any commercial or financial relationships that could be construed as a potential conflict of interest.

## Publisher’s note

All claims expressed in this article are solely those of the authors and do not necessarily represent those of their affiliated organizations, or those of the publisher, the editors and the reviewers. Any product that may be evaluated in this article, or claim that may be made by its manufacturer, is not guaranteed or endorsed by the publisher.
